# Book Reviews

**Published:** 1816-03

**Authors:** 


### CRITICAL ANALYSIS
### *OF RECENT PUBLICATIONS*
IN THE
### DIFFERENT BRANCHES OF PHYSIC, SURGERY, AND MEDICAL PHILOSOPHY.

---

*Cases of Diseased Bladder and Testicle; illustrated with Etchings.* By WILLIAM WADD, Surgeon. 4to. pp. 72. Callow.

WE have already had occasion to notice the accuracy and perspicuity of Mr. Wadd, in his Treatise on Strictures, in which he has revived Mr. Hunter's practice, at the same time rendering his language more intelligible, and compressing his subject. If he has added other improvements, it must be admitted that he had an advantage over his predecessor, in the number of intermediate publications, many of which might have been spared if Mr. H. had been well understood and fairly treated; but their appearance has now placed the question of caustic in so many points of view as to render it readily comprehended by the attentive reader.

The present performance claims our particular notice, not only on account of its intrinsic merit, but for the novelty of a work illustrated by the graphic art, in a manner which must supersede all doubts as to its meeting the precise intentions of the author. This will be best explained by transcribing part of the preface.

"The study of surgery has been greatly facilitated by the introduction of engravings to illustrate morbid changes. Preparations, wet or dry, however beautifully executed, are, from the time of their completion, gradually losing their value, by loss of colour, change of figure, and even from the very delicacy of their materials. Add to this, their utility is much lessened by the circumscribed spot in which only they can be examined. In the circle of the theatre they relieve the lecturer, but arrive at many of the hearers when the subject for which they were introduced is passed, and from that period can be only imperfectly recollected, if they are not entirely forgotten. Engravings, on the contrary, may be multiplied to any number, and are always accompanied with minute explanatory references, whilst morbid preparations require a constant interpreter.

"It must, however, be admitted, that inaccuracy in the figure or reference may be productive of errors, of which the common artist, taught only to study effect, is a very imperfect judge.

Hence the difficulty of procuring satisfactory anatomical representations, even from the ablest masters.

"The early habit of pencilling morbid appearances of sufficient interest to deserve notice, has, by degrees, furnished the author with a large collection of drawings. Of these, when, in compliance with the wishes of his medical friends, he has been desirous of offering to the public a selection of the most interesting, he has always been discouraged by the difficulties above-mentioned. On this subject he had frequent conversations with his friend Mr. Hills, whose philosophical pursuits and pre-eminent talents as an artist are well known. This gentleman not only advised the author to undertake what it would be difficult to explain or correct in others, but, as a further encouragement, offered his own instructions, to enable him at once to secure and multiply the productions of his pencil, by means of the etching needle. Such a proposal from one who, unrivalled in the peculiar department of his art, has, in the execution of a work representing the character of living animals, surpassed the productions of this or any other country, was eagerly adopted, and may serve as an apology for the attempt.

"On a review of the sheets, the name of the late Mr. Hunter very frequently meets the eye. Though this was unintentional, it was also unavoidable; nor is it easy to conceive how others who have gone over the same ground can have escaped the same repeated introduction of that *Clarum et Venerabile Nomen.*"

From the nature of the work, it will be easily conceived that, without the accompanying plates, the greater part of it will be uninteresting, if not unintelligible. The author's object was minute accuracy, and to accomplish this he has availed himself of every means in his power. Hence it would be doing great injustice to his diligence were we to mutilate his work by giving any part of it in an imperfect form. We shall, therefore, transcribe some of the introductory chapters, giving an account only of the contents of the rest.

"*Diseases of the Bladder and its Appendages.*—Diseases of the urinary organs are among the most distressing of those which do not necessarily shorten life. The complicated structure of the male organs readily accounts for this, and renders a minute attention to every part, their respective functions, and the causes by which they are interrupted, of the utmost importance. Yet Mr. Hunter is the first, and almost the only person who has given us a rational and systematic view of the various changes in which difficulty in performing a necessary function embitters the life of so many, from its middle period to its termination. His remarks, though invaluable, were only intended as an appendage to his history of a disease, with the consequences of which most complaints in these parts were, till his time, confounded. They are consequently short, but were found quite sufficient for most practical tical purposes, till Sir Everard Home introduced, as an almost universal remedy, the caustic, which that great master had reserved for the most desperate cases. The application of a general remedy implied an uniformity in the cause of the disease; and Sir Everard's book consisted only of histories of cures, without due attention to that arrangement which formed a new epoch in the study of these complicated complaints. It ought, however, in justice to be remarked, that Sir Everard, in his later publication, appears to have lost much of his former partiality for severe and uncertain remedies.

"Mr. Hunter has also given us a perfect and plain description of the gradual enlargement of the prostate gland, in many subjects at an advanced period of life. It is not easy to conceive what should have induced his relation, pupil, and successor, to consider a diseased enlargement as a natural formation—at a time when, as M. Condorcet remarks, anatomy has arrived at its acmè, 'when every thing which the eye of the observer, assisted by the microscope, has been able to discover, is already ascertained'—could it be believed that a part should have been overlooked by all former anatomists in a gland so often and so minutely examined. If the high authority of his master, and, I may add, almost the only master acknowledged in the present day, was insufficient, the candid report of his own pupil might have convinced Sir Everard of his too hasty conclusion. Happily this error has led to no severity of practice, nor is it likely, nor even that the authority of Mr. Hunter's successor should induce its adoption in our anatomical schools.

"In the description of the diseases of this gland, I have, therefore, retained Mr. Hunter's, and the established doctrine, concerning its original form and subsequent changes."

The first chapter contains the history of two cases which, as Dr. Baillie remarks, are somewhat rare—Calculi in the prostate gland. The history during life (the great desideratum in Dr. Baillie's otherwise invaluable collection of morbid engravings) is added; and the addition of the plates renders the whole as complete as such accounts can be made. The subject is continued in the succeeding chapter. In the third, we have a masterly representation of an enlarged prostate, with a portion projecting into the bladder, and in a state of ulceration. The fourth plate describes a bladder in a most wretched ulcerated state: the history subjoined is particularly interesting. The next is still more so, as being more minute. In this the prostate was enlarged, the bladder thickened, and the surface covered with coagula. In the history of the case, the author recommends injecting into the bladder, and gives some important directions for the manner of doing it. We pass over several others on account of the following history, the theory of which

 has has not, we think, been sufficiently reduced to proof, though among the maxims of Sydenham.

"The subject was about sixty-five years of age, had strictures, and fistulæ in perinæo; but the urethra was sufficiently open to allow the greater quantity of the urine to pass off that way, a part only coming through the fistulæ. He was very subject to the gout, and occasionally, during a fit, would be seized with strangury and retention of urine, which, notwithstanding the diseased state of the prostate, could always be relieved by means of a small gum catheter. His death was occasioned by an accident.

"That gout sometimes induces a disordered action of the urethra and bladder, is generally admitted; and the following cases seem to confirm it. The first was in a gentleman of most temperate and regular habits, who, till that time, never had a complaint of any kind in the urethra, or any of the urinary organs."

Subjoined to the descriptions and representations of diseased bladders is an account and etching of some cancerous appearances about the nates, of calculi passed by the female urethra and cut from the male, with a diagram of the two largest calculi in Britain found in the human bladder. The account of diseases of the testicles is introduced by a warm compliment to Mr. Hunter on the manner in which he has reduced the laws of sympathy to some order; and also on the remarks of Mr. Abernethy, and the illustrative cases by Mr. Ramsden.

A chapter follows on Hydrocele, which contains some new matter, and some most important theories, confirmed by actual dissection of subjects whose history was known during life. As Mr. Wadd informs us, in his dedication to Sir James Earle, that he was for ten years his apprentice and pupil, and as, during that period, the mode of cure by injection was first introduced, this passage cannot fail to interest all our readers: we have, therefore, transcribed as much as our limits will permit.

"Whilst (says our author) I had the honour of visiting with Sir James Earle, there was scarcely an operation of any kind performed by him at which I was not present; and, as to Sir James we owe the fortunate revival of this important operation, with its present improvement, it will be supposed that the cure of the hydrocele by injecting the tunica vaginalis testis, made a very considerable part of his practice, and gave me an opportunity of seeing it under every form. In some cases, after caustic, seton, incision, external applications, and other operations, have failed, and even where the injection had been previously tried by others less acquainted with the practice, he was fortunate enough to succeed.

"The only instances of failure were two cases, in which an attempt tempt was made at a further improvement in the operation; and a third wherein no irritation was produced, in consequence, as it was supposed at the time, of the servant's having diluted the wine. Each of these were cured by a second operation shortly afterwards. Whether even this was necessary cannot now be ascertained, but is by no means certain.

" 'The proper object,' says Sir James Earle, 'of all operations for the radical cure of the hydrocele, is to produce such an adhesion of the distended vaginal coat of the testis with the gland, or such a consolidation of contiguous parts, as shall annihilate the cavity in which the water constituting this disease is contained.' The same is Mr. Pott's language. 'The cure is accomplished merely by the coalescence of the tunica vaginalis with the tunica albuginea;' and Mr. Sharp, in his Critical Enquiry, tells us, that, 'upon examination of several hydroceles after cure, it appeared evidently it was wrought by an universal adhesion of the testicle to the tunica vaginalis.' Such, I believe, were the sentiments of every surgeon of eminence, till Mr. Ramsden ventured to dissent from the established doctrine, asserting that the obliteration of the cavity of the tunica vaginalis testis was not essential to the cure, and that it did not happen unless the curative process had been carried to unnecessary severity.

" Among my notes is a memorandum which very much confirms Mr. Ramsden's opinions. A gentleman underwent the operation in May, 1799. He left town at the end of June. The beginning of July he stated by letter, that the hydrocele had returned as large as before the operation; and in the middle of the next month he wrote word that it had entirely disappeared. The operation had, therefore, excited a new action in the parts, and though the effusion of fluid had returned, yet the absorbents had recovered their function.

" That adhesion takes place between the tunica vaginalis and testis, where there has been a certain degree of inflammation, has been repeatedly demonstrated; and that it is the general effect of the usual mode of injection; but, if the cure can be accomplished by less irritation, and without any change in the parts from their original formation, many might be inclined to undergo it, who would not be willing to hazard an operation under any other circumstances.

" Mr. Ramsden has not confirmed his theory by dissection, but brings abundant proof of transparency in the scrotum after the operation; and on that fact its validity chiefly rests. He attached great importance to the ascertaining the transparency of hydrocele, and in all cases made it his first object of enquiry, thinking that the surgeon who neglected this 'grand characteristic' gratified his vanity at the risk of his patient's security.

" The usual injection is two parts wine to one of water, or if the tunics are thin, the testicle enlarged, or any circumstance require caution, it is made of equal parts, wine and water. Even the the latter proportion is sometimes productive of considerable pain and tumefaction.

"With a view of following Mr. Ramsden's plan of curing by only exciting a new action, with as little pain as possible, I have so lessened the quantity of wine, that the irritation produced has been such as not to detain the patient at home after the day on which it was used; and I am inclined to think that very little irritation of the sacculus is sufficient for the cure of most hydroceles, that do not exceed half a pint in the quantity of fluid, nor six months from their first appearance."

This subject is enriched with many accurate etchings, which, though they possess not all the softness of engraving, are sufficient for every purpose of illustration. On the whole, we are so well pleased with Mr. Wadd's performance, that we are anxious to see his example followed, and extended to other branches of morbid anatomy.

---

*Edinburgh Medical and Surgical Journal*, No. XLV. for January, 1816.

(Continued from p. 155.)

Art. V.—*Case of Fracture of the Skull, in which a quantity of Brain was lost, and a real Hernia Cerebri successfully treated by Pressure; and an Account of the good Effects of Tincture of Bay-leaves to Wounds, Ulcers, Burns, &c. and of Charcoal internally in Dysentery.* By C. K. Crawford, Surgeon, R. N.

The title of this article speaks for itself. The subject of the hernia cerebri was a boy betwixt two and three years old. No part of the scalp was lost.

Art. VI.—*Case of very extensive Wound of the Abdomen, with complete Division of the Ileum, and Penetration of the Cavity of the Thorax.* By Thomas Calton, Surgeon, Collingham.

The subject was a boy seven years old, and of a peculiarly placid temper. We mean not by this to undervalue the cure; but, as far as we conceive our duty, to add to its credibility.

Art. VII.—*On Puerperal Fever, as it appeared at Holloway, near London, in the early part of the year* 1812. By David Dunn, Member of the Royal College of Surgeons in London.

We shall spare Mr. Dunn and his friend Dr. F., whoever he is, by passing over the two first cases. It is, however, creditable to the author, that he improved by an examination of the dead bodies. But that a practitioner so near town should never have known the necessity of bold blood-letting in puerperal fever, (which may be called peritonitis,) before these cases occurred to him, nor have read nor heard of it till Dr. Armstrong and Mr. Hey wrote, surprises us a little.

Art. VIII.—*Three Cases of Inflammation of the Heart, with the Appearances on Dissection.* By Andrew Duncan, Jun. M.D. Professor of Medical Jurisprudence in the University of Edinburgh.

The first of these cases occurred in a miner, twenty-eight years of age. We shall give the symptoms and appearances after death in the author's own words, omitting the treatment, which is the less important as it was unsuccessful.

"Complains of severe head-ach, and a gnawing pain in the region of the stomach, not increased by pressure, but much aggravated on taking food; with a sense of a ball in the under part of the abdomen, working upwards through the course of the alimentary canal to his throat, when he feels as if immediate suffocation would ensue. This feeling he has experienced almost every night for nearly three months past. Frequently, although not always, at the beginning of each paroxysm, he has a very copious flow of urine, approaching nearly to the colour of table-beer. During the fit his belly swells, and it is always relieved by a great discharge of flatus by the mouth. He is not sensible of foaming at the mouth during the paroxysm; nor, if standing, is he thrown to the ground, but becomes so giddy as not to be able to stand without assistance. He also complains of great debility, want of appetite, interrupted sleep, and a disagreeable taste in his mouth on awakening in the morning. Has a delicate look; pulse 104, of moderate strength; heat natural; tongue covered with a brownish crust, with a clean edge; some thirst; belly costive, in which state he always feels worst; urine varies; when his complaints are severe, it is increased in quantity, and of a higher colour.

"Began to complain about three months ago. Was first seized with the pain and sense of tightness in the region of the stomach. Can remember no cause for his complaints, except being exposed to cold and hard work. Nothing happened at that time particularly to affect his mind. Says he has used stomachic bitters and bark, without alleviation of complaints. Was formerly troubled with symptoms nearly similar to the above, but never to such a height. Was able always to follow his occupation, and enjoyed a good appetite."

"*Sectio Cadaveris.*—The contents of the cranium, thorax, and abdomen, were carefully examined.

"About an ounce of a transparent and colourless fluid was found accumulated between the dura mater and arachnoid coat, about the lower surface of the cerebellum, and the origin of the spinal chord, obviously collected here in consequence of the position sition of the head after death, and, in all probability, partly derived from the spinal canal. Within the lateral and middle ventricles, there was also from half an ounce to an ounce of a serous fluid. The brain and its membranes presented no appearance of disease. The lungs adhered very extensively on both sides to the parietes of the chest, but chiefly on the right. The adhesions were close and strong. The substance of the lungs had undergone no morbid alteration of structure. The pericardium contained from six to eight ounces of a greyish muddy serum, with flakes of a curdy matter. The heart had suffered a change of texture, not less singular than extensive. The whole parietes of the right auricle, and the anterior side of the corresponding ventricle, were found converted into a greyish-coloured substance, of a very uniform texture, and having the consistence nearly of the prostate gland. There was not the slightest vestige of any of the natural tunics of the heart in this substance. The parietes of the left ventricle had undergone a similar change for about an inch all around its auricular orifice, and there was fully a third of the septum between the ventricles, towards the base of the heart, converted into the same substance. Between the arterial orifices too, the septum was a little thicker than usual. The inner surface of the right auricle was covered with a sort of efflorescence, not unlike that which is often exhibited by hydatid cysts. The left auricle exhibited no morbid appearance. The valves, tricuspid, mitral, and semilunar, were of their natural structure, as also the roots of the pulmonary artery and aorta.

"Within the cavity of the abdomen there was most extensive disease. The whole mesenteric glands were enlarged, so as to form one irregular mass. When divided, they exhibited an appearance like that already described, as having been found in the sides of the heart. The whole of the colon had its coats thickened and rendered exceedingly tender. At various points of it, there was a vascularity accompanying this thickening, which indicated inflammation. At other parts, the sides of the intestines had become considerably thinner, and projected in the form of little pouches, which adhered to the parts with which they happened to be in contact. The whole of the jejunum, and a great part of ileum, were in the same state. In the angle formed by the junction of the transverse part of the colon with the large curvature of the stomach, which were found closely applied to each other, there was a stratum of half an inch broad, and a quarter thick, of the same sort of substance as was found in the heart. Towards the surface of the liver, in its right lobe, and at its upper convex part, there was a knot of the same kind of matter, of the diameter of a shilling; but, in other respects, this organ had a healthy appearance. There were two or three nodules of a similar matter, in the substance of the pancreas. The spleen presented nothing remarkable.

"There was about a pint of serum effused into the cavity of the the abdomen, which, except where it had touched the gall-bladder, and in consequence acquired the colour of bile, was of the usual yellowish tint, and quite transparent."

A long series of reflections, by the author follows: some of these we shall notice.

"This case appears interesting,

"1st, From its rapid and unforeseen event.

"2d, From the appearances discovered on dissection.

"When admitted, it was supposed to be a case of hysteria in a male,—a rare occurrence, yet not unobserved. Some consider hysteria and hypochondriasis as the same state of disease, modified by sex; the former being peculiar to women, the latter to men. This is, in general, true, but exceptions do occur; and it is not sufficient to reject all the testimonies adduced in proof of this, because we ourselves have not seen it. Unequivocal cases of hysteria in men have been seen by Dr. Trotter and others. Hypochondriasis certainly oftener affects females.

"The difference between them depends much on temperament. Hysteria is peculiar to the sanguine, and hypochondriasis to the melancholic, temperament. In hysteria, the affections of the mind are characterised by fickleness and mobility; in hypochondriasis, by the obstinacy with which the mind broods over a single subject. In hysteria, the patient is violently affected by every circumstance external to herself, and is agitated by every sudden impression; but the paroxysm is no sooner over, than she laughs at her own folly, thinks no more of it, and resumes her wonted spirits. In hypochondriasis, on the contrary, external circumstances make little impression on the patient; and if a sudden and violent impression rouse him for a moment, he speedily returns to brood over his loss of health, or finds in what has just passed fresh cause for despondency.

"The symptom in Baxter giving rise to the idea that his disease was hysteria, was the accurate description he gave of the globus hystericus. But after he came under our care, the symptom did not recur.

"I was then led to consider his complaint as hypochondriasis, from the pain of stomach, increased on taking food; the irregular state of his urine; the costive belly; flatulence; interrupted sleep; bad appetite; foul tongue, and bad taste in his mouth; and still more by his constant complaints. I therefore left him a day or two to observe the symptoms."

We would only remark, that hypochondriasis is a very general, and, unfortunately, a very ill-defined word. In common language, the man had all the symptoms of hypochondriasis; but we regret exceedingly that a professor should be so led away by a nosological term, as to leave so interesting a case for a *day or two*. We wish we had a closer description of this "*efflorescence*, not unlike that which is often exhibited by hydatid cysts." Were they cysts, or what was the difference? No one will question that there was disease enough to kill a man, or that the whole was formed before the author of the paper had any interview with the patient; but it would be very desirable to know whether these appearances were the effect of violent inflammation at a remote period, or had gradually grown during the last complaints. We are well aware of Corvisart's distinctions between the acute, sub-acute, and chronic inflammation of the heart; the great objection to which is, that the two latter are, we fear, often the consequence only of the first, which was violent enough to produce effects that must prevent the future regular functions of the heart, but not sufficient to produce immediate death. This is a question of the utmost importance, inasmuch as its decision would lead to a due consideration of arresting, at all events, and in its earliest stage, high inflammation in vital parts, without waiting to determine whether we have carditis, pericarditis, or diaphragmitis, or whatever other *itis* we may meet with in nosological tables or dictionary writers.

Dr. Duncan produces some very judicious references to Morgagni and others, concerning diseases in the cardiac system. The only one to his purpose is the case of the Venetian woman, which makes the 23d article of the 44th epistle. This case is highly important on another account, which is only slightly hinted at in this place, but which we shall notice the first time a question occurs concerning the uncertainty of the signs of life.

"She was dissected (says Dr. D.) ten hours after death, and Morgagni, having doubts that it had actually taken place, proceeded with the utmost caution, after having made all the usual experiments to ascertain it."

The doubt which embarrassed Morgagni and his friend arose from the body remaining warm; a question which we are astonished has never occurred to the writers on medical jurisprudence, and which we are still more astonished has never been attended to by the various experimenters and inquirers into the causes of animal heat. But of this more when a proper opportunity offers.

Some judicious remarks follow on the texture of muscles, and the changes they undergo with a view to form a similar analogy regarding the muscular part of the heart. We must not, however, pass over one instance of scepticism concerning a question highly important in this and all other inquiries concerning the heart.

"It is not yet ascertained (says our author) upon what the red colour colour of the flesh of man, and the higher orders of animals, depends. For the fact, that red muscles become white by washing, does not prove that the red colour depends upon the blood, but only that the colouring matter is soluble in water. Fishes have red blood, and yet many of them have white muscles; and, in some animals, different muscles differ very much in colour. This is especially remarkable in the black cock, where the outer layer of pectoral muscles has a very dark colour, and the inner is white, so that, in cutting out a slice of the breast, we have flesh of two very different colours, and yet supplied with the same blood, and even from the same arterial trunks."

In answer to this, we shall only say, in the words of Mr. Hunter, that in all quadrupeds most of the muscles are red. That this redness arises from the blood, is proved by the muscles being red in proportion to the quantity of blood they contain, which is in proportion to the quantity of use required of them. This is evident in all the muscles with whose actions we are acquainted, and is particularly striking in the heart, whose action, being constant, requires the most blood, and in all red-blooded animals it is found the reddest part: next to this are the sphincter muscles, whose actions are also constant, though not to so great a degree. It is not less certain that muscles lose the redness they have acquired in proportion as they are less used. This is an important consideration in the heart, as we shall presently see.

"We find (continues Dr. D.) muscles altered, not only in their colour, density, and cohesion, but even in their very nature. We find them converted into fungous or scirrhous masses, or other diseased composition, or into cartilage, tendon, bone, &c. substances not in themselves morbid, but here unnatural.

"A change of this kind had taken place in Baxter's heart. The greater part of it had lost its natural red colour, and was almost white. This in itself is here a mark of disease; for, although, in different individuals, the intensity of the red colour of the heart differs naturally, as well as the colour of other muscular organs, yet the natural colour does not exceed certain limits; and when there is great and strikingly morbid differences between different parts of the same muscle, it seems always to proceed from disease.

"But it was not merely in colour that the deviation from the natural composition of this heart was evident. Its texture or grain was also evidently changed, for, in that portion which had lost its natural colour, the fibrous arrangement natural to it was everywhere obscure, and in some places not to be discovered, even with the aid of a lens. It was not, however, absolutely destroyed, because, by boiling, it not only became hardened and crisped, though in a less degree than the natural fibres, but I succeeded, by very long boiling, in perfectly developing its fibrous

 texture, texture. In short, the change of texture may be best described by saying, that, in a considerable portion of this heart its grain had become much finer than natural; so fine, indeed, that, without the assistance of art, the fasciculi upon which the grain of muscles depends could not be recognized.

"This change was dispersed through different parts of the heart, but was chiefly obvious in the right auricle, septum, and left ventricle. In general, the change was less perfect near the inner surface, so that there the flesh, though white, was still visibly fibrous; while, as it approached the outer surface, its fibrous arrangement disappeared altogether. Yet, in the midst of these cartilaginous-like portions, a small bundle of red fibres was occasionally intermixed.

"As it is highly improbable that an alteration of structure of this kind took place in the course of the few days during which Baxter declined so rapidly, we may suppose that it is not inconsistent with the heart carrying on its functions with tolerable regularity.

"To ascertain this point, however, we should compare the phenomena which have occurred in other cases of the same kind,—but here we altogether fail; for, so far as I have been able to discover, no case exactly resembling this, in the kind of alteration of substance, has been observed, unless, perhaps, that which I have translated from Morgagni.

"It is not unusual to observe white opaque spots upon the surface of the heart, sometimes thicker, and sometimes thinner; sometimes more, sometimes less distinct; and, at first sight, this heart seemed to present a spot of this kind, larger, thicker, and more distinct than usual; but, on cutting into it, the difference was obvious, for these spots are owing to the formation of a false membrane exterior to the proper coat of the heart, so that it can be removed, and leave the heart covered with its proper tunics. This appearance occurs so often, that Baillie and Soemmering do not consider it as being morbid. In Baxter's heart, however, it appeared that the white spots were owing to the substance of the heart itself being altered in composition."

"The next morbid appearance (proceeds our ingenious author) noticed in Baxter's, was a sort of efflorescence, covering the inner surface of the right auricle, not unlike that which is often exhibited by hydatid cysts. It consisted of globular and pedunculated masses, attached to the parietes, and had very much the appearance of fatty particles; but on analyzing them by fire, they were evidently fibrine or albumen, at least when placed on hot iron they did not melt, but shrivelled and burnt with the smell of feathers.

"The question in regard to these is, whether they existed during the life of the patient, or were first formed by the gradual coagulation of the blood after death, and the concretion of its coagulable lymph with the sides of the auricle. But, besides occurring in the auricle, it appeared that a similar substance, but more in the form form of a membrane, had lined the right ventricle, at least fragments of it, passing under the columnæ carneæ, still remain in the prepared heart.

"Except in general form, these have a considerable resemblance to the polypi of the heart, about which it was so long, and is still, disputed, whether they were formed before or after death; and probably the same arguments may apply here, as the only difference is, that, in the usual form of polypi, there are one or more long fibrous-like masses of considerable size, often entering into the mouths of the vessels. That the great majority of these are formed after death, there cannot be a doubt; indeed there are few bodies in which they are altogether wanting; but still there seem to be incontrovertible proofs of their being occasionally formed during life. The most decisive instance of this that I have met with, has been admirably described in this Journal, by my friend, Mr. W. Wood. Indeed, I am inclined to think that the loose body, as well as the attached mass, observed by him, had the same origin with what Dr. Gordon and myself have denominated the efflorescence in Baxter's heart. At any rate, they prove the possibility of its having existed there long before death, which becomes more probable when we consider, that such an appearance would be often observed, if it originated in the coagulation and separation of the blood in the heart after death, as in this there can be no great diversity. The great objection to this opinion is the natural state of the circulation during life; but this admits of explanation, as no part of this efflorescence was loose, or situated so as to obstruct the flow of the blood.

"There are three ways in which such concretions may be formed: 1. By an organized growth from a surface of greater or less extent. 2. By an inflammatory exudation from a living surface, such as takes place often on the lungs, and sometimes on the outer surface of the heart itself. And, 3. The coagulation of blood, in consequence of a portion of it being removed out of the current of the circulation, and becoming stagnant.

"In the first manner are formed tumours, warts, &c.; and sometimes even loose bodies, by their subsequent separation, when originally attached by a narrow neck; but this could not be the process in the present instance."

On this subject we have, of late, so amply delivered our opinion, in our remarks on Mr. Hodgson's very ingenious and well-conducted inquiries concerning the diseases of arteries, that we shall only refer our reader to them. Indeed we hardly know how to account for Dr. Duncan's passing over Mr. Hodgson's labours in this part of his reflections. All further remarks we shall preserve for the conclusion of the article.

Of the second case we shall transcribe the passages most important to pathology; the death of the patient in this, as in in the former, rendering the therapeutic part less interesting.

"Mary Rickman, æt. 22, married.—March 11, 1815.

"Complains of cough, attended with considerable expectoration; pain in the thorax, more particularly referred to the sternum and between the scapulæ, increased by full inspiration and cough; and of difficulty of breathing, aggravated by exertion. She is unable to lie in the horizontal position.

"Complains also of pain in the right haunch, increased by pressure and exercise, but unattended by swelling. There is also considerable swelling of both ankles, which pits slightly on pressure; but there is no discolouration of the skin, nor is there any pain. Pulse 88; H. moderate; tongue rather white; much thirst; appetite bad; B. costive; catamenia irregular; urine natural; sleeps ill.

"Complaints first began about ten days ago, with shivering and flushing; pain in the breast and cough, followed the next day by pain in the shoulders, elbows, knees, and right haunch; but the pain in all these joints, with the exception of the last, is now gone.

"Has been bled, and taken purgatives and diaphoretics, but with little benefit.

"Attributes her complaints to exposure to cold.

"Mitt. sanguis ad ℥ xij. pleno rivo.

"App. vesicat. parti affect.

"12th.—Was bled last night; no buffy coat on the blood, which, however, is tolerably firm. The blister applied to her side rose very well; but, as she was not relieved this morning, she was ordered to be bled again, but would not submit to the operation. Has a very severe cough, which distresses her much; and she has had no sleep for eight nights past. Has had no stool. The pain in the side, where the blister was applied, is relieved, but it is more severe in her breast. Pulse 88, rather full, and somewhat hard."

This plan was continued or altered, as circumstances required, till the 18th of the following month, when the patient died; and the examination of the body will readily account for the want of success, however judicious the treatment might be.

"*Sectio Cadaveris.*—On laying open the thorax, a considerable quantity of fluid was found effused into the cavities of the pleura, on both sides.

"The lungs on both sides seemed quite healthy, and of their natural colour; but, on endeavouring to raise them up, they were found to adhere firmly to the pericardium, through its whole extent; to the diaphragm; and laterally to the pleura costalis, by membranous bands; and the lobes were firmly attached to each other, so that they could not be separated without some difficulty.

"The pericardium was found adhering so firmly all round the heart, heart, that it was with difficulty raised from it. It was very much thickened, and evidently consisted of three laminæ; the middle one being opaque, white, and dense; the outer very unequal in thickness; and the inner very vascular, red, and pulpy.

"The heart itself was a good deal larger than natural, and was also thickly covered with coagulable lymph, which, owing to its being torn asunder from the pericardium, appeared very irregular on its surface. Under this lymph, about two-thirds of the thickness of the heart had been changed into a substance somewhat resembling in its appearance condensed fat; and was found in fact to contain fat, as it swam in water, melted, and stained paper with a greasy stain: the remaining third had almost lost its muscular appearance.

"The columnæ carneæ in both of the ventricles were larger than natural. Ossification had just commenced in the semilunar valves, at the mouth of the aorta; but the mitral valve was thickly beset with osseous matter."

Among the reflections of the author, we shall only select the following paragraph.

"Pericarditis is indeed said to be a very obscure disease. And yet, except for my having at first taken up the erroneous notion that it was pleuritis, it was sufficiently obvious in the present instance. We had fever characterizing active inflammation, and the seat of the pain sufficiently indicated the place. Now, the only organs which could be affected there were the pleura, mediastinum, or pericardium. The cough and expectoration, and the absence of symptoms usually enumerated as characteristic of carditis, misled me in my judgment. Rickman had not the *pulsus inequalis, palpitatio et syncope,* of Cullen; nor the constant vomiting of Darwin; nor the palpitation, faintings, quick and unequal pulse of Sauvages; nor the very intense thirst of Burserius; nor the hydrophobia of Daniel; nor the delirium of Davis."

So much for nosology; so much for dictionary writers, systematizers, and even for those who consider themselves practical writers. It is not our intention hastily to condemn either; but to teach the reader how uncertain the best of them must be, and how necessary he will find it to judge for himself.

"The dissection of this case (adds our author) presented a most striking instance of universally and violently inflamed pericardium; and I think I am warranted in considering the inflammation of the pleura in the vicinity, causing agglutination of the neighbouring lobes of the lungs, and effusion into the cavities of the chest, as an effect of the original pericarditis, in consequence of the inflammation spreading by contiguity. The pericardium, in this woman, adhered universally to the heart by a layer of coagulable lymph, without any effusion of serum. Cases of this kind are not always necessarily fatal, for, whenever we find the pericardium adhering to the heart after death, we may infer, that it is the consequence of pericarditis at some former period. This is not not a very rare occurrence. I have myself seen it in a man who died apparently of asthma, and Corvisart has a chapter on the subject. But the most remarkable circumstance in this woman's heart was its partial conversion into fatty matter. Of that I was assured, not only by its appearance, but by chemical experiments, which were perfectly conclusive as to the fact. Corvisart himself never saw an example of this conversion, but says, that it has been seen by some modern anatomists, although their observations have not been published. He also confesses his ignorance of the manner in which such a transformation takes place. In the present instance, we find it accompanying pericarditis, but it does not follow that it was an effect of the inflammation. It may have existed before the commencement of the fatal disease. I do not know that any connection has ever been traced between the conversion into fat of other muscles, and preceding inflammation.

"The commencing ossification of the valves, in so young a woman, is, however, an argument in favour of some state of inflammation having existed in the heart on a former occasion; and the membranous bands, causing lateral adhesions of the lungs, were evidently the effect of pectoral inflammation some time before the fatal attack."

We come now to the third case contained in this interesting and important paper.

"John Macleod, æt. 17, sailor.

"January 25th.—Is affected with pain in the right side, on deep inspiration or on cough; which last is pretty severe, and attended by a very bloody sputum. The chest feels hot but not oppressed. Has head-ach, nausea, retching, and an occasional vomiting of a slimy black matter. Thirst; tongue whitish and moist at the edges. No appetite; B. regular; no sleep; pulse 92, small and soft.

"Complaints of three days duration. Sputum became bloody yesterday morning. Was immediately bled to considerable extent; and a cathartic was administered."

The patient lived, under various treatment, for sixteen days.

"On opening the thorax next day, few or no adhesions were observable; nor was there any considerable effusion of serum into the sacs of the pleura. A little to the right of the sternum there extended from the lower part of the left side a dirty chocolate-coloured bag, which, on being opened, proved to be the pericardium adhering to the lungs, thickened and much distended; also containing two pounds six ounces of perfectly formed pus.

"The inner side of the pericardium, and that part which is reflected over the heart, were covered with a thick coating of a substance resembling condensed curds: and in some parts to a greater depth than others.

"The substance of the heart was very much paler than usual. It had no other peculiarity.

"The dissection of this case shewed that it was an example of pericarditis, nearly as free from complication as it can occur; and, on this account, it becomes the more valuable, as Corvisart informs us that he is in possession of no proper case of acute pericarditis without complication. But here, while we have a very high degree of inflammation of the pericardium, both where it invests the heart itself, as its outer membrane, and where it is reflected as a loose bag around it, there was almost no affection of the neighbouring parts.

"The leading symptoms were at first pain in the right side; certainly a very anomalous symptom, but it must be observed, that the pericardium extended to the right of the sternum; cough, with bloody expectoration; head-ach; nausea; retching; and occasional vomiting; pulse 92, small and soft. In the progress of the disease, the pain became general over the left and lower parts of the heart; the retching continued; the pulse became quick, feeble, and intermitting; and delirium came on. The bloody expectoration was for a short time suspended, and then increased so as to amount to hæmoptysis. At first he complained of want of sleep, afterwards he slept or dosed a great deal. His cough and dyspnœa fluctuated, but increased towards the close of his life, which took place on the fifteenth day from the attack.

"These cases seem to me to form a valuable addition to our knowledge of this interesting disease. In Baxter we have an example of chronic and occult carditis, without any symptom occurring to indicate the affection of the heart; while after death the effusion of coagulable lymph, both into its cavities and into the pericardium, and the change its substance had undergone, left no doubt as to this important organ having been generally affected with inflammation. In Rickman and Macleod, we have cases of acute or subacute pericarditis; the former, accompanied by effusion of coagulable lymph, would have produced, if the patient had survived, a general adhesion of the pericardium, and the latter terminating in an enormous, and necessarily fatal, effusion of pus. The symptoms during life were no less different than the appearances after death; but any attempt to connect the one with the other would be premature."

The few remarks we shall offer will be less necessary for those who have perused with attention our analysis of Mr. Hodgson's performance. We shall, therefore, only repeat, that all the appearances described in these cases prove high inflammation in the early stage of the diseases, at which time only they might probably have been all relieved.

It was unfortunate for all these patients that they were seen so late in the disease by the judicious author of the paper; otherwise, it is at least probable, that all the calamitous events which followed might have been prevented, and, to render the paper practically useful, this, we conceive, should have been the object constantly kept in view in every part of of the detail. That the symptoms were those of debility in the end is evident by the wine directed in one instance, and the wine-whey in the other. But whence, it should be enforced on every young practitioner, did this debility arise, and what would have been the means of preventing it? The answer is ready. By checking the inflammation in the first instance, and thus preventing the irrecoverable condition of so important an organ.

The only additional remarks we shall make on the various appearances are:—that flocculi on the inner surface of the heart or larger arteries rarely appear unless the internal surface has been destroyed by previous inflammation; that there is reason to believe coagula on the sides of these parts when smooth, particularly if uniting an organ and its investing capsule, will, if the patient survives, gradually elongate so as to give an increased scope towards the original motion of these parts on each other; that the pieces of coagulum found, whether in the heart or arteries, arise from the high disposition which is found in the blood to coagulate in the living body, when parts in the neighbourhood are highly inflamed. Such is the true theory of Mr. Hunter's adhesive inflammation. The opinion that such coagula are formed by the stagnation or slow motion of the blood within the heart or arteries, is erroneous, because such a cause in the living body is not equal to such an effect as Mr. Hunter has proved experimentally in several trials. The opake spots on the heart are frequently seen, and when we know the history of the patient may be always accounted for by some previous inflammation.—See *Sir Everard Home's Account of the Examination of Mr. Hunter's Body.*

The fatty appearance or change of the muscle to adipocere can only be explained at present in one way. Inflammation may be carried to the point of mortification. As we rarely find suppuration from mere inflammation in muscles, and never we believe hitherto in the heart, the mortified portion must remain without being separated; and in this state, having lost all the properties of life, it becomes in the condition of those muscular substances which have been for some time kept from the air in moist situations: that is, it is convertible into adipocere.

Whilst we have made these very free remarks on Professor Duncan's paper (all of them, we assure him, confirmed by the examination of cases under our own inspection,) we are free to acknowledge the obligations we owe him; and earnestly recommend him to pursue such inquiries in private families, where he can with more certainty learn all the previous symptoms.

Art.

Art. IX.—*Case of Inguinal and Popliteal Aneurism, cured by tying the External Iliac Artery.* By William Newbiggin, Esq. President of the Royal College of Surgeons, Edinburgh.

The success of this case does credit to the improvement of modern surgery, and the consequent increased courage of the practitioners.

---

*Commentaries on some of the most important Diseases of Children.* By John Clarke, Esq. M. D. &c. &c. &c. Part the First. 8vo. pp. 198. Longman and Co. 1815.

A work on the Diseases of Children, from a practitioner of Dr. Clarke's age, experience, and celebrity, cannot be perused without interest. After filing, if we may be allowed the expression, all his practical observations, by several years' public lecturing and frequent consultations, we might expect a concentrated series of aphorisms, which the young practitioner should always keep in view. If we are disappointed in this respect, it is, probably, because this meritorious physician has not lived to complete his labours. We must, therefore, be thankful for the volume as it is; and only hope that his surviving relation will, from the notes of the deceased, and his own observations, favour the world with that accumulated knowledge we have a right to expect from such sources. This is the more to be desired, because the Preface informs us that in a future part it is the author's intention to enter more at large on medicines proper for children—"to lay before the reader some account of those medicines which, in the course of long experience, have been found to be most useful in the various disorders of children."

The first chapter contains some general observations on the diseases and mortality of children, and on the state of medical knowledge on these points. Here the author begins by regretting, in common with all other medical men, the imperfection of our bills of mortality, which, however correct in the number and ages of the persons buried in the districts, conveys very little medical information, on account of the imperfect manner in which the diseases are expressed, and the ages at which each proves fatal. Another imperfection, continues our author, is, that it contains no information as to births; so that it is impossible to ascertain the relative proportion of births and deaths. Dr. C. does not seem aware that the same difficulty attends the burials, many being interred in dissenting cemeteries, which afford no account to the parish clerks, as the opinion of the searchers is no-where

 registered.

registered. This is evident by the language of those documents, which only mention burials, and in what parish-churches, or parish burial-grounds.

From the bills, however, imperfect as the are, the author draws the following conclusions, taking the gross numbers for the last forty years of the preceding century.

"It appears from this table of the burials, as published in the bills of mortality annually, that in forty years the whole number of burials amounts to 836,285.

"It appears that of this number 281,408 died before they had attained the age of two years; and that, of the surviving 554,877 (after deducting those who died under two years of age), 113,393 died before they reached the age of ten years.

"If the assumption above stated be true, that in early life the births and burials are equal, then it follows, that of all the children born within the district comprehended in the bills of mortality, nearly a fourth die under two years of age; and of the survivors, about a fifth in the succeeding eight years, that is, under ten years of age.

"In some years, as in 1760, 1765, 1766, 1767, 1768, 1769, and many others which may be easily referred to, more than a third of all the burials are of children under two years of age.

"The result of this examination of the yearly bills of mortality in London and the suburbs, is very important, not only in a medical, but in a political view. It must on all hands be admitted that, cæteris paribus, the strength of every nation will be proportionate to its population, and that every thing which has a tendency to diminish the population, must affect its strength and prosperity. It has been maintained, and perhaps with great truth, that the population of a country will be proportionate to the means of subsisting the inhabitants:* but the want of subsistence can hardly be supposed in this country to be a sufficient explanation of the extensive mortality among children under ten, but especially under two years of age. It is a subject of infinite importance to the community, and no measure should be neglected, which might have the effect of checking this great mortality in the early periods of life."

---

"* It is very difficult to set bounds to the power of a country to subsist its inhabitants. In Great Britain, and still more in Ireland, there are large districts of waste land unemployed at present, so that they could maintain a greater number than they do. In Ireland, immense tracts of bog are capable of being reclaimed, if encouragement were given to the inhabitants to exert themselves. Moreover, the commercial intercourse of these dominions with other nations would produce the importation, if necessary, of very large quantities of grain in the way of exchange, if the population should increase much beyond its present extent."

"There

"There are some diseases," continues our author, "of which (with a very few exceptions) the human constitution is susceptible once only in the whole of life: such are the hooping-cough, the measles, small-pox, and, perhaps, the scarlet-fever, attended with the ulcerous sore throat.

"Children in early life will be very liable to these diseases, especially in large towns, which are seldom entirely free from them, and where, therefore, in some seasons, and in certain unknown constitutions of the air, or from different degrees of susceptibility in the human constitution at different times, they occasionally become endemic, and prove mortal to a very great extent."

After these judicious observations, we were surprised to hear so experienced a writer recommending, or rather enforcing, separations, to prevent the spreading of diseases, "from which large towns are seldom free." How, we would ask, can a person promise himself a moment's security from dangers he never can see or prevent, and of which, till he is secured, he must live in perpetual terror."

"It is (we are told) in the power of the legislature to establish such a system of police upon the subject of contagious disorders, as to lessen considerably the extension of some of them, by making provision for separating the sick from the healthy, at least in most cases of contagious disorders. This might be effected by establishing public hospitals at the national expence, for admitting the poor, without interest or recommendation, when labouring under diseases capable of being communicated by contamination, and allotting separate establishments for different diseases.

"With respect to the small-pox, this separation of the healthy from the diseased might be made compulsory upon all ranks of society. In the natural or casual small-pox, all infected persons should be compelled to be separated from the healthy. If poor, they would be glad to take advantage of a public establishment, by which their expences would be diminished, and where food and medical attendance could be had gratuitously. Those families whose superior station in society would not make it necessary or desirable to take advantage of such institutions, should be compelled to avoid all general communication with the healthy part of the community; to inscribe on their houses, in large legible characters, that the small-pox is there; and to perform a reasonable quarantine after the termination of the disease."

And when all this is done, we would ask, what is accomplished? Can any persons promise themselves a moment's security, but by those means which they are more likely to adopt in proportion as the danger of infection is the greater? This subject has, however, been so often canvassed, that we forbear to follow the author through the rest of the chapter, till, at the close, he touches on a subject so entirely within his own sphere, that we cannot do otherwise than offer it in his own words.

"Important as the attention to the diseases of children confessedly is, no part of medicine (to use no stronger expression) has been so little cultivated.

"In all the works of all the best writers, from the age of Hippocrates to the present, inclusive, scarcely any practical information is to be found upon the subject, unless a few scattered and detached observations may be so considered.

"The reason of this is sufficiently apparent, when it is recollected, that the practice of midwifery among the ancients, in all nations whose history is known, was committed to women only, and those generally of the most ignorant description.* From this cause, it would naturally follow that the treatment of the complaints of children, as well as the general regulations of their diet, &c. would devolve upon a class of persons utterly incompetent to make, far less to record, any useful observations. Ignorance is generally presumptuous, and they would soon think themselves competent to act as physicians in the diseases of children, and little disposed to ask assistance from others.† Hence the ancient physicians were deprived of the means of procuring information; and the deficiency of it in their writings is, therefore, less a matter of surprise.

"In this country the practice of the healing art is divided between physicians and surgeons; the latter of whom, till of late years, were very little acquainted with medicine. Women were principally employed in the practice of midwifery; and surgeons were called to cases of mere mechanical difficulty, and seldom saw the patient afterwards.

"The establishment of the Royal College of Physicians appeared very likely to remedy the prevailing ignorance of the persons practising midwifery, (as the members of it, by their charter, have a right to practice both surgery and medicine,) and to enlarge the knowledge of the diseases of women and children.‡ But there seems to have been a fatality on this point, and that women

---

"* Terence, in his Andrian, thus describes the midwife: 'Sane pol! Ista temulenta est mulier & temeraria.'

"† Etsi vero nonnulli existimant infantium morbos solum naturæ & mulieribus, quæ infantes tractant, committendos esse; tamen & hic medico suæ partes sunt. Sennertus.

"‡ One of the greatest advantages proposed by this institution was that of examining and admitting into their own body, or licensing, all persons who proposed to practise medicine in the limits of their jurisdiction, touching their skill, after examination by the president and censors, all of whom were supposed to be themselves of good education, and of sufficient age and experience to qualify them as judges of the fitness of candidates."

and and children should not have a reasonable chance of relief in the diseases of child-birth and of early life.*

"It would hardly be believed possible in a civilised land, if it did not stand recorded in the bye-laws of the College, that any persons, at any time, could have had sufficient influence upon so learned a body (and who were, therefore, less liable to prejudice) to induce a majority of them to accede to a prohibitory bye-law by which the Fellows of the College are compelled to exclude themselves from practising midwifery, and, therefore, from acquiring much knowledge of the diseases of infants and children. It seems to be a law calculated for the perpetuation of ignorance, by preventing men of the best education and the highest attainments in learning from adding to the stock of medical knowledge on subjects most dear and important to society."

The second chapter is on the structure of the mouth and organs of digestion in children, and the diet proper for them at different times. The most important part of this is what relates to wet-nurses, and the mortality of their own children, whilst the foster-child is so carefully preserved.

"As the matter, however, stands at present, it is hardly a question whether society at large is a gainer or loser by the employment of hired wet nurses. If the child lives for which the wet nurse is invited, by the prospect of present gain, to forsake her own, the child of the wet nurse often dies, or it becomes diseased or crippled. Her other children are neglected, and her husband, for want of her society, becomes drunken and profligate: she rarely returns home contented with her former station, but compares her present privations with the indulgencies which she has left: the whole comfort of the labouring man's fire-side is broken up, and society has only exchanged the life of one child for that of another, with all the disadvantages above enumerated.†

"On

---

"* The immortal discoverer of the circulation (in whose praise an oration is annually delivered in the College of Physicians) appears, from passages in his works, to have practised midwifery himself, and therefore feelingly expresses himself on the point of the low state of midwifery in his time. 'Melius profecto cum pauperculis res agitur, iisque quæ furtim gravidæ factæ clanculum pariunt, nullius obstetricis advocata opera.'—Harv. de Partu."

"† From the consideration of the prodigious mortality of the children of wet nurses,* Dr. Denman, and some other physicians and surgeons, practising midwifery, of whom the writer was one, a few years ago, endeavoured to establish an asylum where the children of wet nurses might be received and nursed, and voluntary subscriptions were very liberally made for the support of it; but

"* In some families six, in others eight, wet nurses had lost their own children."

"On the whole it would be better, perhaps, that the children of the wealthy should be brought up artificially where the mother does not suckle, because they would have every advantage of good nursing, cleanliness, air, and medical treatment, and would, therefore, have a better chance of living than the child of the wet nurse, who will want all these advantages."

This is among the many kind attempts of benevolent people to do a wonderful deal of good, and to seek for sources overlooked by others. Are there not children of poverty enough already; and are parents so careless of their offspring likely to bring them up with the same advantages to society as those who, from affection, interest, or ambition, are anxious to live a second time in their posterity? But, if the lives of these unhappy creatures are at all events to be preserved, let it be by a tax on the wealthy, who, by their large and tempting bribes, deprive the natural offspring of their true birth-right. Let no person of fortune engage a wet nurse without paying a certain sum to an establishment where the child, thus deprived of its proper food, may at least have a vicarious mother. Dr. Clarke might have extended his moral remarks a little further, and in this religious age have descanted on the immorality of preferring the mothers of base-born children, whose fathers are not likely to be troublesome, thus giving encouragement in vice and misery!

A valuable chapter on Dentition follows. This commences, like most works of practical importance in the present day, with a reference to Mr. Hunter, who, we are told, has given all the information necessary or useful concerning the process of dentition. Those, therefore, who wish for a scientific knowledge of the subject, are, of course, referred to the grand source of all modern pathology. The author's own remarks, we need not say, are judicious, but we are obliged to add that they cannot be compressed.

The next subject which attracts our author's attention is convulsions, "a vague term," as he observes, "comprehending a number of diseases," arising from irregular action in almost every part of the bodies of infants. The disease is very accurately described, and the more common causes pointed out; after which we have some valuable practical remarks on a peculiar species of convulsion in very young children. One general conclusion, drawn from the whole, is,

---

but it was found that the expenditure was too great to be supported by private munificence; and the projectors had the mortification of being compelled to relinquish the plan altogether, for want of sufficient means to uphold it.

is, that in every case of convulsion, be the cause what it may, the brain is at the time organically affected either directly or indirectly. This chapter is the more valuable, the subject being illustrated with some very apposite cases. The treatment of these complaints follow, in which the causes are very properly enlarged upon as the means of directing our practice. The whole concludes with a proper caution against the use of unknown compounds, or quack medicines in general.

A very important chapter follows on Phrenitis, or Inflammation of the Brain; a complaint certainly much more common than has till lately been suspected, and even at this time too often overlooked, or passed over under the unfortunate term typhus, or some such expression, undefinable, but easily retained by the nurses. All the symptoms are most correctly described, and the high sensibility of the subject in the early stages of the disease is nicely contrasted with the subsequent torpor. This cannot be too much insisted on, as by parity of reasoning we may easily understand how, in the early stage of chronic hydrocephalus, the child is often found superior in intellect to its equals in age. We cannot follow our author through the whole of this chapter, which may be said to contain a complete history of the disease, and the mode of treatment, so free from all adventitious matter, that it becomes impossible to compress it more than the author; and to offer a partial quotation, without any introductory matter, would not be doing justice to such a composition. There is a note, however, which, being detached, we may venture to transcribe; and it is with much reluctance that we feel obliged at least to question the accuracy of Dr. Clarke on a subject, the causes of which he has had so many opportunities of tracing, and has so well availed himself of all.

"Squinting (says he) in all cases, is probably owing to some pressure upon the origin of the nerves supplying the muscles of the eyes, and not, as some have imagined, to the two eyes not having the same focus. It does not appear how this should paralize the retracting muscles, or give an increase of strength to the adductor muscles, either of which is equivalent to the other.—In the evolution of the brain, or the growth of the basis of the skull, however, some irregularity in the degree of pressure on the nerves may arise, which may affect the muscles to which they belong.—Squinting is certainly, like many other disorganizations, hereditary; and the writer has known instances where children of squinting parents first shewed a disposition to squint at nearly the same age as the parent had done, and this several years after birth, without any symptom of oppressed brain having occurred.

In such cases it may be presumed that the pressure is entirely partial."

It is not from any disposition to attack the only weak point of a well-digested whole, (the too common error of our fraternity,) that we offer the following remark; but we conceive what we are going to offer particularly necessary, because it refers to a practical caution within the management of any one, but especially incumbent on the medical attendant. Children who, for a length of time, have had one eye only inflamed, which has, in consequence, been constantly protected from the light, contract the habit, from necessity, of viewing objects with only one eye. If this has happened at an early age, the sympathy between the eyes is not so confirmed as to produce a constant motion in one corresponding with the other: the inflamed eye will become gradually motionless, and, when the inflammation has ceased, the habit of looking at objects with one eye will continue, and the recovered eye will remain motionless. In this case, squinting arises entirely from the different situation of the two globes, and is easily cured by obliging the young patient to keep his head fixed, whilst he is directed to view an object on the side of the fixed eye. At first he attempts to see it with the moveable eye, but, finding that impracticable, if his curiosity is artfully excited, he will at last bring the fixed eye into motion, and the other, retaining its sympathies, will follow. There is a very good paper in the Philosophical Transactions on this subject. We have introduced it, not to show our superior attention to the subject, but as an illustration of another part of this valuable chapter, in which the author shows, in a pointed as well as most candid manner, the means by which some kind of paralytic limbs may be recovered. Speaking of limbs paralyzed in consequence of effusion during inflammation, Dr. C. remarks,

"Though it is contrary to the practice pursued by many mechanics, it appears to the writer to be best to make the support on the inside of the leg, by which means the flexor muscles will be prevented from acting together, and this will give a better chance for a more equal antagonization of the two sets of muscles, than if the support is made on the outside, in which case the peronæi will have nothing to do, and (like other inactive muscles) they will lose the little power which they before possessed.—For the same reason, no more support should be given than is barely sufficient to produce the effect.

"If no means are employed early, the parts will accommodate themselves to their new unnatural state, and no art can replace them afterwards, in the greatest number of cases; yet the writer has known an instance where a man, who had been a cripple from his his childhood, was enabled, by machinery, to walk with a stick, after he had attained the age of puberty. Much will depend upon the exertion of the patient in all such cases, and applying the mind intently to the paralysed limb, by which means the power of action may be sometimes regained, even in cases apparently hopeless.*"

The few passages we have selected, and even the few objections we have started, will show the care with which this chapter has been compiled. As it contains the most common of all the acute, and frequently incurable, diseases of infants, we cannot be sufficiently thankful for the remarks of so valuable a writer. The whole leads to the consideration of such diseases as are the usual consequence of the various stages of phrenitis, if the early symptoms should not prove fatal: these are idiotism, paralysis, and epilepsy. For the reasons above assigned, we shall not attempt to shorten, or mutilate by extracts, the valuable matter contained in this concluding chapter of the FIRST PART.

After what we have said, it must be unnecessary to recommend the work: we shall conclude as we began, by expressing our earnest wish that the second part may appear as soon as possible.

---

### MEDICAL AND PHILOSOPHICAL INTELLIGENCE.

"* This idea first occurred to the acute mind of Mr. John Hunter."

